# DPP4 inhibition curbs systemic inflammation

**DOI:** 10.1186/s13054-025-05599-x

**Published:** 2025-08-15

**Authors:** Katharina E. M. Hellenthal, Sebastian Kintrup, Timo Wirth, Laura Brabenec, Christina Cursiefen, Rebekka Beulen, Katharina Hollmann, Martin Lehmann, Philipp Burkard, Johannes Roth, Klaus Schughart, Luisa Klotz, Jan Rossaint, Patrick Meybohm, Nana-Maria Wagner

**Affiliations:** 1 Department of Anesthesiology, Intensive Care and Pain Medicine, University Hospital Muenster, Muenster, Germany; 2 Department of Neurology with Institute of Translational Neurology, University Hospital Muenster, Muenster, Germany; 3 Department of Physiology and Pharmacology, Karolinska Institutet, Stockholm, Sweden; 4 Department of Anaesthesiology, Intensive Care, Emergency and Pain Medicine, University Hospital Würzburg, Oberdürrbacher Straße 6, 97080 Würzburg, Germany; 5 Institute of Immunology, University of Muenster, Muenster, Germany; 6 Department of Microbiology, Immunology and Biochemistry, University of Tennessee Health Science Center, Memphis, TN USA; 7 Institute of Virology Muenster, University of Muenster, Muenster, Germany

**Keywords:** Vasculature, Inflammation, Capillary leakage, Cardiac surgery, DPP4 inhibitor

## Abstract

**Background:**

Systemic inflammation is a critical clinical condition regularly observed in the context of surgery-induced trauma or infection. Systemic inflammation induces an ubiquitous activation of the vasculature and vascular dysfunction related to organ damage and adverse outcomes. The dipeptidyl peptidase-4 (DPP4) modulates the receptor preferences and activity of a multitude of humoral substrates mediating the systemic inflammatory response. We here determined whether DPP4 inhibition is a means to beneficially modulate systemic inflammatory responses affecting vascular and organ integrity.

**Methods:**

In cardiac surgery patients medicated with DPP4 inhibitors, we used a systems biology approach for in-depth characterization of the perioperative immune response and assessment of macro- and microvascular dynamics compared to control patients. In parallel, we mechanistically evaluated the efficacy of DPP4 inhibition on modulating immune responses, capillary leakage, vasoplegia and endothelial transcriptomic profiles in mice with severe systemic inflammation.

**Results:**

Preoperative oral intake of the DPP4 inhibitor sitagliptin modulated innate and adaptive immune phenotypes and was associated with augmented microvascluar integrity, reduced vasoplegia and improved clinical parameters of capillary leakage in patients undergoing cardiac surgery. In mice, DPP4 inhibition curbed the inflammatory response to a polymicrobial sepsis resulting in a massive reduction in endothelial gene activation assoicated with preserved vascular barrier function, augmented vasopressor responses and organ integrity.

**Conclusions:**

We conclude that DPP4 inhibition may be a safe and potent means to curb immune responses to surgery or infection, resulting in a preservation of vascular integrity that translates into organ protection and improved clinical outcomes.

**Trial registration:**

https://www.clinicaltrials.gov; Unique identifier: NCT05725798, study start: 2022-02-01.

**Graphical abstract:**

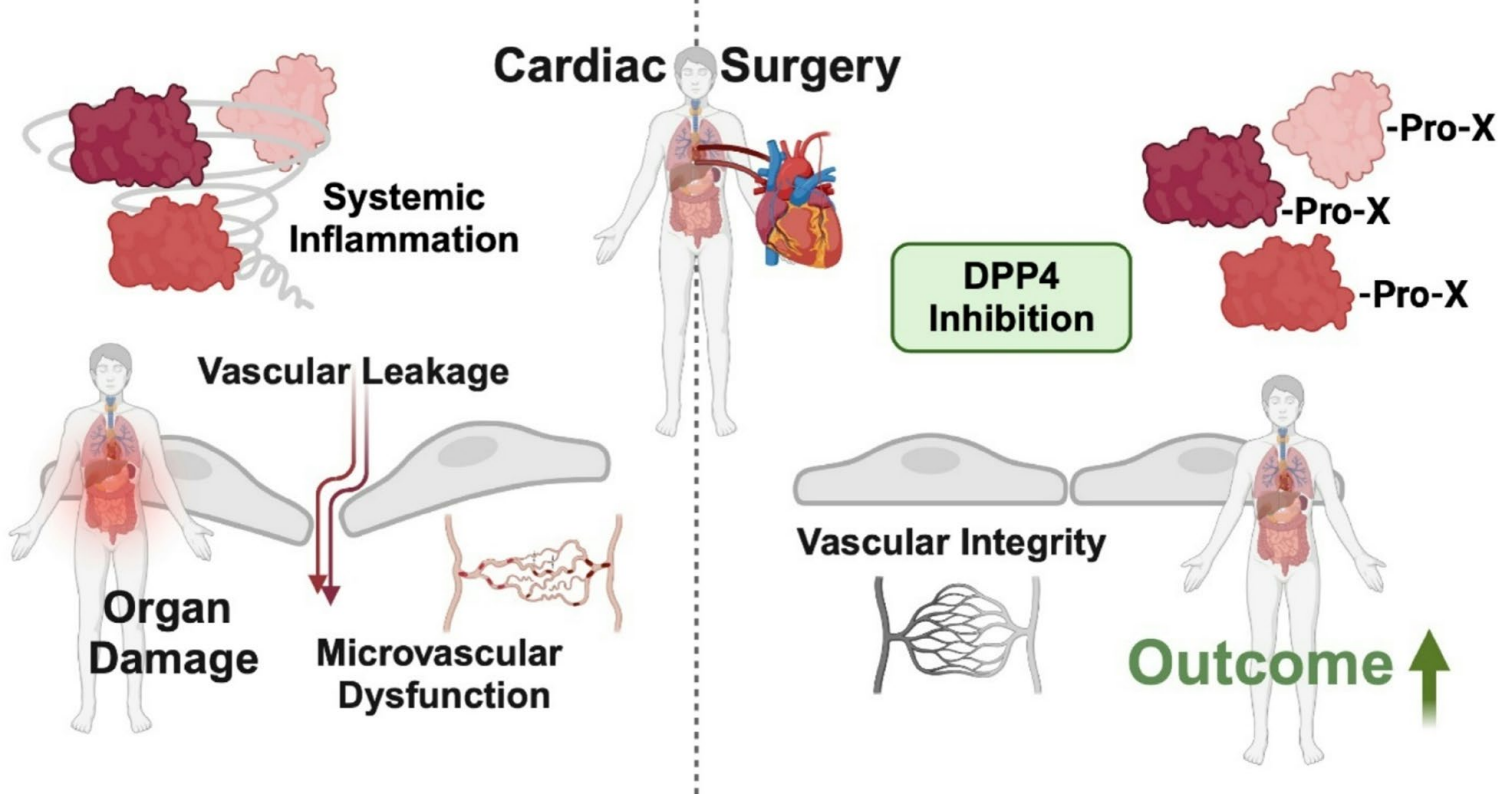

**Supplementary Information:**

The online version contains supplementary material available at 10.1186/s13054-025-05599-x.

## Background

A systemic inflammatory response is regularly observed after major surgery or during sepsis-associated bacteremia. In these scenarios, immune cells get activated by molecular patterns of cellular molecules liberated upon tissue injury or microorganisms. Activated immune cells release pro-inflammatory mediators and interact with the endothelium, rendering it into a pro-inflammatory phenotype promoting adhesion and extravasation of immune cells, opening of otherwise tight inter-endothelial junctions and pro-thrombotic processes [1, 2]. This loss of endothelial barrier integrity facilitates shift of fluid to the interstitium leading to tissue edema formation and compromised microcirculation due to an increase in interstitial pressure [3]. Intravascular fluid depletion leads to hypovolemia and hypotension, often amplified by vasoplegia requiring high doses of vasopressors [4]. These profound abnormalities of the micro- and macrohemodynamics impair perfusion and oxygen supply to tissues, endanger cellular integrity and promote organ dysfunction. Although organ dysfunction and failure are the primary causes of morbidity and mortality perioperatively and during sepsis, there is still no therapeutic strategy available to ameliorate the dysregulated immune response in systemic inflammation.

Various humoral pro-inflammatory mediators released during the systemic inflammatory response, such as interleukin-6 (IL-6) or tumor necrosis factor (TNF)-α, exhibit a highly conserved penultimate proline at the N-terminus that is involved in regulating receptor preferences and activity [5]. Dipeptidyl peptidase-4 (DPP4), originally known as lymphocyte cell surface protein CD26, is a regulatory enzyme catalyzing the removal of N-terminal dipeptides, preferentially with a proline in the N-terminal position. DPP4 is expressed on the endothelium, leukocytes and circulating soluble and critically affects the biological activity of its substrates, for example IL-6 [6, 7]. DPP4 inhibitors, also known as “gliptins”, have been added to treatment regimens of type 2 diabetes, as they strongly reduce DPP4-mediated inactivation of the incretin hormones glucagon like peptide-1 (GLP-1) and gastric inhibitory polypeptide (GIP) and thus lower blood glucose levels. In addition, gliptins are associated with reduction of oxidative stress, vascular dysfunction and anti-inflammatory effects. Long-term DPP4 inhibition exerts protective properties on cells and tissues, such as reducing oxidative stress, inflammation, fibrosis and apoptosis, promoting regeneration and ameliorating endothelial dysfunction [8]. However, the summative effect of DPP4 inhibition during acute systemic inflammation has not been evaluated so far.

Using a systems biology approach, we here perform in-depth characterization of the perioperative immune response in cardiac surgery patients during DPP4 inhibition. We correlate these results to measures of vascular function in patients after cardiac surgery and confirm the modulatory potency of DPP4 inhibition during systemic inflammation in septic mice.

## Methods

### Experimental design of the clinical study

For this prospective observational clinical study, *n* = 14 patients (female and male) scheduled for elective on-pump-cardiac surgery were recruited and written confirmed-consent was obtained. All surgeries took place at the University Hospital Münster between February 2022 and May 2023. Exclusion criteria were acute or chronic infections, type 1 diabetes mellitus, immunological disorders, immunosuppressive therapy, off-pump-surgery and emergency surgery. Medication was continued according to current recommendations [9], including sitagliptin 100 mg. Patients without sitagliptin medication were recruited as controls. A total of 36 mL EDTA-anticoagulated blood was drawn on the morning of the first postoperative day. Peripheral mononuclear blood cells (PBMCs) were isolated by density gradient centrifugation and stored pending analysis in the vapor phase of a liquid nitrogen tank. Data on macrohemodynamics and laboratory parameters were obtained from the patient data management system. Patient management and data analysis were performed blinded to the treatment conditions.

### Flow cytometry

Cellular immune characteristics were determined by flow cytometry. For this purpose, PBMCs were thawed in a 37 °C water bath for 8 min. The cell suspension was transferred to a 50 mL conical tube and 9 mL pre-warmed Roswell Park Memorial Institute (RPMI)-medium [RPMI (Sigma Aldrich), 10% fetal calf serum (FCS) Gold Plus (BioSell), 1% Glutamax (Gibco), 1% Na-Pyruvate (Invitrogen)] was added prior to centrifugation at 300 g for 10 min. Supernatant was discarded and cell pellet was resuspended in RPMI-medium. PBMCs were counted and viability was assessed using a Countess II automated cell counter (Invitrogen). Subsequently, PBMCs were subjected to immune phenotyping by flow cytometry. For this purpose, PBMCs were directly stained with fluorochrome-conjugated antibodies directed against lineage-defining epitopes, markers of cellular differentiation, activation, and maturation, as well as receptors involved in proliferation and regulation of effector functions and measured using specialized flow cytometry panels, as previously described [10]. In addition, intra-cellular/-nuclear epitopes were investigated by incubation of PBMCs with Perm/Fix buffer (BD Biosciences) at room temperature for 20 min and subsequent staining at 4 °C in Perm buffer (BD Biosciences) for 30 min. Functional capacity of immune cell subsets was investigated by both, unspecific stimulation with PMA (polymethacrylate)/Ionomycin/Brefeldin A (leukocyte activation cocktail, LAC, BD Biosciences) and specific stimulation by re-directed cross-linking of CD3 or DNAX Accessory Molecule-1 (DNAM-1) and 2B4 followed by staining for lineage markers, as well as cytokines or CD107a, a marker for the directed degranulation of cytolytic vesicles. For detection of monocyte derived cytokines, PBMC were stimulated with 100 ng/mL LPS (E. coli O127:B8, Sigma-Aldrich, Cat. No: L4516). Samples were acquired on a CytoFlex flow cytometer (Beckman Coulter) under daily quality control by CytoFlex Daily QC Fluorospheres (Beckman Coulter) and analyzed using Kaluza Analysis 2.1 (Beckman Coulter) and OMIQ (Omiq, Inc.) software with gating strategy, as previously described [10].

### Human cytokine measurement

Serum samples of patients drawn 18 h after surgery were subjected to analysis using a human inflammation 20 plex-panel (ProcartaPlex, ThermoFisher) analyzed with a Luminex LX 200-device for the detection of perioperative cytokine release.

### Hemodynamic management

In the postoperative period on the intensive care unit, to maintain the mean arterial blood pressure target of at least 65 mmHg, patients were subjected to 500 ml of crystalloid infusion after a positive passive leg raise test [11]. If negative, patients were subjected to continuous norepinephrine infusion.

### Analysis of microvascular function

Immediately after surgery at admission to the intensive care unit (ICU), as well as 18 h postoperatively, sublingual microcirculation was assessed by incident darkfield microscopy using a hand-held video microscopy camera (CytoCam, Braedius Medical B.V.). Microcirculation includes arterioles, venules and capillaries with a diameter < 20 μm. At least three recordings per patient, originating from different sublingual regions, were obtained by gently putting the video microscope on the sublingual mucosa without causing pressure artifacts. According to the consensus guidelines on the assessment of sublingual microcirculation, each recording was motion-free, evenly illuminated, well focused and had a length of 5 s [12, 13]. Furthermore, saved recordings were software-corrected using “CytoCamTools V4” to minimize image movement. Video analysis was performed manually using the software “Capillary Mapper 1.4.5”. Proportion of perfused vessels (PPV) and microvascular flow index (MFI) were the parameters we primarily assessed. PPV is a grid-based binominal score defined as the percentage of perfused vessels per total number of vessel crossings. MFI also represents a semi-quantitative grid-based score per quadrant assessing the blood flow velocity from 0 (stop flow) over 1 (intermittent flow) and 2 (sluggish flow) to 3 (normal flow) per quadrant. The percentage of fluid area was determined in at least three screenshots per patient of the videos of the sublingual microcirculation originating from different independent sublingual regions by black-and-white-color thresholding (white ≙ vessel-free area ~ edema, black ≙ vessel area) and area measurement on Image J (Version 1.51r, National Institutes of Health, USA) and the mean value was calculated for each patient. All recordings were obtained and analyzed by the same experienced investigator, blinded to the experimental conditions [14].

### Experimental design of the murine sepsis model

DPP4 knockout mice were purchased from Taconic Biosciences (Germany, Model No. 11085, C57BL/6-*Dpp4*^*tm1.1Mrl*^). C57Bl/6J mice and DPP4 knockout mice (on B6N background) were housed in the central animal facility under pathogen-free conditions with food and water ad libitum and with a 12-hour light/12-hour dark cycle. For C57BL/6-*Dpp4*^*tm1.1Mrl*^ animals, C57BL/6 N were used as controls. Female and male 10–12 weeks old mice were used for experiments in equal amounts. Polymicrobial sepsis was induced by cecal ligation and puncture (CLP), as described previously [14, 15]. Briefly, after ensuring adequate anesthesia by intraperitoneal injection of ketamine (100 mg/kg) (Narketan^®^, Vetoquinol, France) and xylazine (10 mg/kg) (Rompun^®^, Provet AG, Schwitzerland) solved in sodium chloride (5 mg/kg), the murine abdomen was shaved and disinfected with 70% ethanol. Afterwards, median laparotomy was performed by a 1–2 cm incision. Following the ligation of the distal cecum with a 4 − 0 suture 14 mm distal to the tip, the cecum was punctured twice using a 25G canula. After replacement of the cecum into the abdomen, the incision was closed. After surgery, mice were kept warm on a heating plate at 37 °C during recovery. 5 mg/kg body weight sitagliptin (Tocris), DPP4 inhibitor K579 (Tocris) or sodium chloride as vehicle control were injected into the tail vein directly after surgery or with 6 h time delay. Sham operated control group underwent the same surgical procedure without ligation nor puncture of the cecum.

### Murine immune cell counts and cytokine multiplex analysis

For determination of murine immune cell counts (white blood cells, lymphocytes, monocytes, platelets) 18 h after sepsis induction, heparin-anticoagulated whole blood was analyzed using a Sysmex hemacytometer according to the manufacturer’s instructions. To decipher humoral characteristics of the ongoing inflammation, murine plasma was withdrawn 18 h after sepsis induction and cytokine and chemokine multiplex analysis was performed according to the manufacturer’s instructions (Cytokine & Chemokine 36-Plex Mouse ProcartaPlexTM Panel 1 A, Thermo Fisher). Microplate was analyzed in a Luminex LX 200 analysis device.

### Assessment of capillary leakage

To visualize edema formation of murine intestines, 10 mg/kg bodyweight Evans blue (Sigma) dissolved in 0.9% sodium chloride were injected 17.5 h after sepsis induction by CLP or sham surgery and respective injection of 5 mg/kg of the DPP4 inhibitors sitagliptin (Tocris) or K579 (Tocris). 30 min later, mice were sacrificed and murine intestines were put on silicon filled dishes. Representative pictures showing Evans blue content of murine intestines were taken using iPhone X. For histological assessment of edema formation and organ integrity, murine lung, liver and kidney were removed, kept in 4% formaldehyde at 4 °C, processed with Microm STP 120 Spin Tissue Processor (Thermo Fisher) and embedded with paraffin. 6 μm thick sections were made by using Microm HM 355 S (Thermo Fisher), then transferred to a microscope slide and incubated at 37 °C in order to dry. Five representative pictures per mouse were taken using Lionheart FX microscope (BioTek, Software Gen5 Version 3.05) by a blinded observer. Pulmonary edema was analyzed by ventilated pulmonary area using Image J (Version 1.51r, NIH). Histopathological changes in murine liver were scored based on the criteria congestion, edema, infiltration of immune cells and necrosis [16]. Tubular injury of murine kidney was quantified by presence or absence of epithelial necrosis, loss of brush border, cast formation and tubular dilatation [17]. Wet/dry ratio indicating edema formation was analyzed in murine lung, liver, kidney and intestines after 7 days of incubation at 37 °C and weight measurement on a precision balance.

### Pressure myography

For the assessment of vasomotor function, third-order mesenteric resistance arteries were dissected 18 h after sepsis induction and pulled on glass cannulas in a vessel chamber (Living Systems Instrumentation) in calcium-free buffer (10 mM 4-(2-hydroxyethyl)−1-piperazineethanesulfonic acid (HEPES), 140 mM NaCl, 5 mM KCl, 1.2 mM MgCl_2_, 10 mM glucose, 1 mM EGTA), as previously described [18]. Experiments were performed in calcium-containing buffer (Ca-free buffer without ethylene glycol-bis(β-aminoethyl ether)-N, N,N′,N′-tetraacetic acid (EGTA) including 2mM CaCl_2_). After development of a stable myogenic tone at 80mmHg, constriction response to 10nM (*R*)-(−)-phenylephrine hydrochloride (Phe, Tocris) was assessed using digital video edge detection.

### Murine endothelial cell isolation and transcriptomic analysis

Dissociation of lung tissue into single-cell suspensions for subsequent cell separations was performed using MACS Technology (Miltenyi Biotec) using gentleMACS octo dissociator with heating units (Miltenyi Biotec, #130-096-427) and lung dissociation kit (Miltenyi biotec, #130-095-927), following depletion of CD45^+^ cells (Miltenyi biotec, CD45 MicroBeads, #130-052-301) and enrichment of CD31^+^ endothelial cells (Miltenyi biotec, CD31 MicroBeads, #130-097-418) according to the manufacturer’s instructions. Purity and viability of isolated pulmonary endothelial cells were verified by flow cytometry analysis (BD FACSCanto II). Following RNA isolation using RNeasy Mini Kit (Quiagen), RNA was subjected to next generation sequencing. One animal was excluded in the sepsis group subjected to sitagliptin treatment due to insufficient drug delivery. Sequencing was performed at the Core Facility Genomics of the Medical Faculty Münster. Quality and integrity of total RNA was controlled on Agilent Technologies 2100 Bioanalyzer (Agilent Technologies; Waldbronn, Germany). PolyA + RNA was purified from 100 ng total RNA using Poly(A) mRNA Magnetic Isolation module Kit (NEB E7490L, New England Biolabs). The RNA Sequencing library was prepared with NEBNext^®^ Ultra™ II Directional RNA Library Prep Kit for Illumina^®^ (New England Biolabs). The libraries were sequenced on Illumina Nextseq 2000 using NextSeq2000 P2 Reagent Kit. Samples were sequenced in two different batches (batch 2: 238 cycles, paired end reads 2 × 111 Nu with an average of 26.7 M reads per RNA sample and batch 1: 88 cycles, single reads 72 Nu with an average of 26.3 M reads per RNA sample). Reads were first checked for quality with package FastQC (version 0.11.4), then trimmed using Trimgalore (version 0.4.4) with default settings. Trimmed reads were mapped to mouse genome annotation mm11 (ENSMBL Mus_musculus.GRCm39, release 104) using STAR [19], with default settings. Further analyses and visualizations of data was performed using the R software package (version 4.2.1 and 4.4.3, R_Core_Team 2013a) and RStudio (version 2022.07.2 and 2024.12.1). Mapped reads were counted using RsubRead (version 1.32.4) [20]. Raw counts from both batches were combined and then normalized and log_2_ transformed using function rlogTransformation from the DESeq2 package (version 1.16.1) [21] and an increment was added to the normalized values to make all values positive. For identification of differentially expressed genes (DEGs) raw counts from both batches were combined, a model was used to correct for batch effects (design = ~ batch + group), and then pairs of groups were contrasted. DEGs were identified using a multiple-testing adjusted p-value of < 0.05 and exhibiting more than a 2-fold (log_2_ = 1) difference in absolute expression levels. VENN diagrams were generated with the function vennPlot (http://faculty.ucr.edu/~tgirke/Documents/R_BioCond/My_R_Scripts/overLapper.R). Heatmaps were generated with the function heatmap2 of package gplots (version 3.1.1; https://github.com/talgalili/gplots). Functional analysis of DEGs were performed using the R software package cluster Profiler (version 4.14.6 [22]).

### Culture of human and murine endothelial cells, permeability assay

Human Umbilical Vein Endothelial Cells (HUVECs) were seeded on transwell inserts (0.4 μm pore size, Corning) and incubated for 48 h until confluency. Endothelial cells were pre-incubated with 1 µM sitagliptin (ChemCruz) for 2 h following stimulation with 1 ng/mL DPP4 substrate procalcitonin (PCT) [14, 23], and application of 1 mg/mL 70 kDa fluorescein isothiocyanate (FITC-) labeled dextran (Sigma Aldrich). After 2 h, fluorescence was measured at 520 nm using a plate reader (Tecan, Magellan Pro) in samples taken from lower chambers.

### Transcriptomic analysis of HMPECs

Human Pulmonary Microvascular Endothelial Cells (HPMECs) were seeded on Tissue Culture Plate (6 well) and incubated for 48 h until confluency. Endothelial cells were pre-incubated with 1 µM sitagliptin (ChemCruz) for 10 min following stimulation with 1 ng/mL PCT. After 6 h, endothelial cells were dissociated with Accutase (Sigma Aldrich) and RNA isolation was performed with RNAeasy plus Mini-Kit (Qiagen). Following quality control, samples were subjected to sequencing using a NextSeq 2000 platform with 30 M reads per sample. Data was trimmed using FASTQ with cutadept nd aligned to human reference genome (GCF_000001405.40_GRCh38.p14). Unique- and multimapping reads were used for read quantification and normalized to log2-TPM counts. Differential gene expression was evaluated using GFOLD [24].

### Statistical analysis

Statistical analyses were performed with SPSS 28.0.1.1 and Graph Pad Prism Software version 7 (Graph Pad, USA). Effect was estimated by difference in means and 95% confidence interval and P values ≤ 0.05 were considered to be statistically significant. Data are presented as mean ± standard error of mean (SEM). Statistical significance was determined, as indicated in the respective figure legends. In brief, for the clinical study, normal distribution was tested applying the Shapiro-Wilk test. Depending on distribution, either student’s t-test or Mann-Whitney-test were performed to determine the significance level. For comparisons between three groups, one-way ANOVA/Bonferroni or Kruskal-Wallis test were used. Nominal variables were analyzed using contingency tables and the exact Fisher test. For immune cell panels with prominent sitagliptin-effects, we used dimensionality reduction and clustering for the investigation of complex phenotypic properties within defined lymphocyte subsets. For this purpose, flow cytometry files were loaded to OMIQ (Omiq Inc., Santa Clara, SA; www.omiq.ai), linearized by ArcSin transformation and randomly subsampled to equal cell numbers. Subsequently, unsupervised clustering of single cell data was performed using PhenoGraph (k = 300 (69)) and clusters projected onto a t-distributed stochastic neighbor embedding with Barnes-Hut approximation (bh-SNE) map using differentially expressed markers as input channels. Deviations between the groups were depicted using color coded log_2_-fold changes (log_2_fc) heatmaps superimposed on the bh-SNE-plots. These types of analyzes were performed for the CD4^+^/CD8^+^ T-cell-panel, pro-monocyte-panel and the monocyte adhesion- and migration-panel. Scripts generated in R version 4.0.2 (‘Taking Off Again’) and RStudio version 1.3.959 were used for heatmap representations of dimensionality reduction maps. Additionally, a principal component analysis of all patients of the three study groups was performed using 115 cell surface markers (supplement) (scaled values) that were significantly different between any two groups after a linear regression analysis using limma [25, 26]. For experimental data, after testing for normality distribution, statistical significance was determined by using One-way analysis of variance (ANOVA) followed by correction for multiple testing applying the Bonferroni method or Student’s t-test.

## Results

### Perioperative DPP4 inhibition modulates the inflammatory response to surgery

**Fig. 1:**
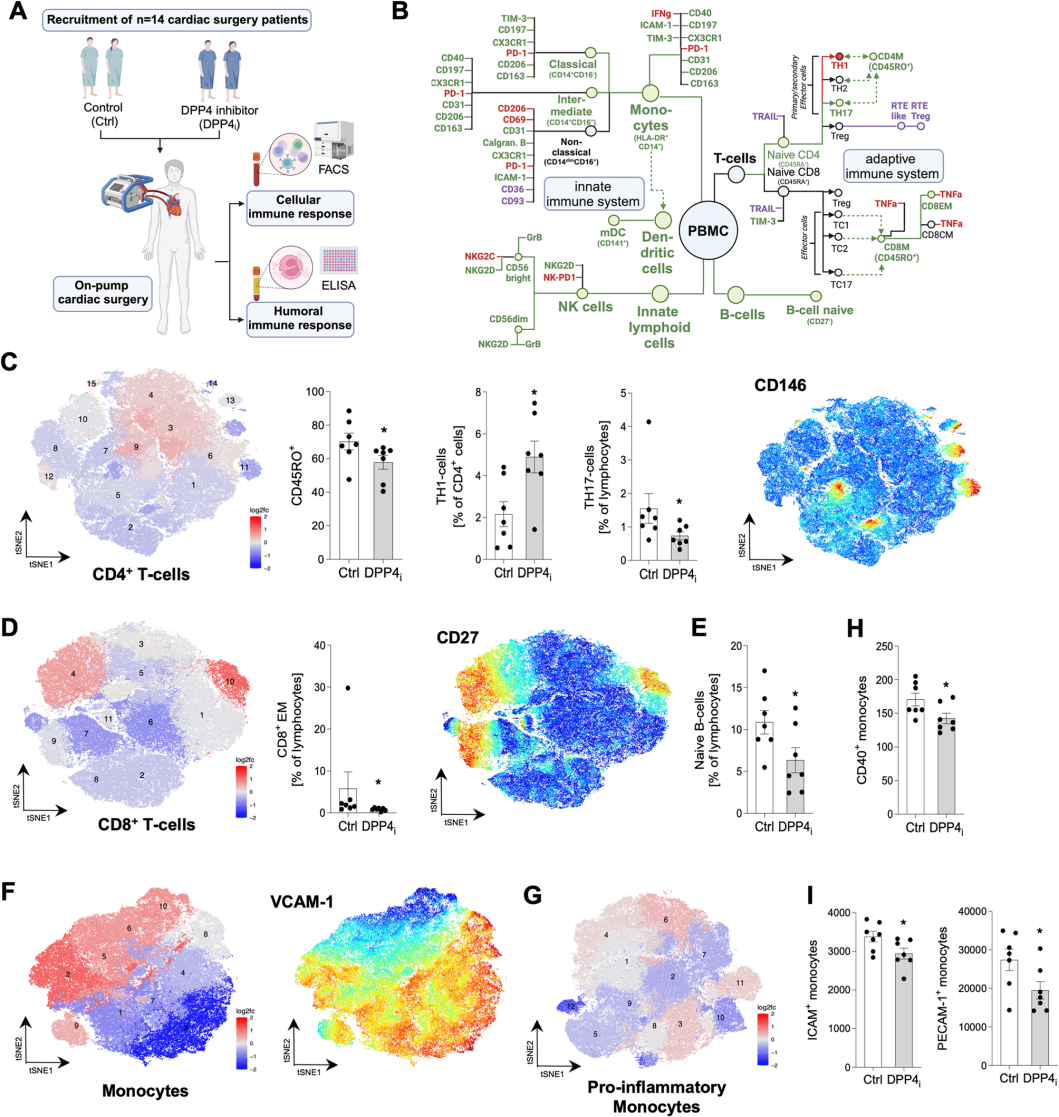
Perioperative DPP4 inhibition modulates the inflammatory response to surgery. **A**: Recruitment of *n* = 14 patients undergoing elective cardiac surgery with *n* = 7 subjected to preoperative intake of the DPP4 inhibitor (DPP4i) sitagliptin. Analysis of the cellular and humoral immune response by flow cytometry and ELISA techniques. Designed with BioRender.com. **B**: Anti-inflammatory alterations induced by DPP4 inhibitor treatment on main PBMC populations, their subsets and surface markers measured by FACS compared to untreated control patients are marked in green, pro-inflammatory alterations in red, alterations with unknown effects in purple. BioRender.com. **C**: bh-SNE representations obtained from concatenated samples (*n* = 7 vs. 7) within the CD4 + panel 18 h after surgery. Quantity of CD4 + CD45RO + cells 18 h after surgery (*n* = 7 patients/group; mean ± standard error of mean (SEM); Mann-Whitney-test). Quantity of T helper 1-cells (TH1-cells) (CCR4- CCR6-CD183 + CD4 + memory T-cells) and T helper 17-cells (TH17-cells) (CCR4 + CCR6 + CD183- CD4 + memory T-cells) 18 h after surgery (*n* = 7; mean ± SEM; TH1 unpaired t-test; TH17 Mann-Whitney-test). CD146 expression within the CD4 + clusters. **D**: bh-SNE representations obtained from concatenated samples within the CD8 + panel 18 h after surgery. Quantity of CD8 + effector memory (EM) T-cells (*n* = 7; mean ± SEM; unpaired t-test). CD27 expression within the CD8 + clusters. **E**: Quantity of naive B-cells 18 h after surgery (*n* = 7; mean ± SEM; unpaired t-test). **F**: bh-SNE representations obtained from concatenated samples (*n* = 7 vs. 7) within the monocyte adhesion- and migration-panel 18 h after surgery. VCAM-1-expression within the clusters of the monocyte adhesion- and migration-panel. **G**: bh-SNE representations obtained from concatenated samples (*n* = 7 vs. 7) within the pro-inflammatory monocyte-panel 18 h after surgery. **H**,** I**: Expression of CD40, ICAM-1 and PECAM-1 on monocytes (*n* = 7; mean ± SEM; unpaired t-test). For the bh-SNE representations, unsupervised clustering by PhenoGraph identified cell clusters, as indicated by numbers. The color of the cluster signifies the log2-fold changes of cluster abundance in up- (red) or downregulation (blue) of the DPP4 inhibitor group compared to the control group. Asterisks indicate differences vs. control group (*p < 0.05)

Patients undergoing on-pump cardiac surgery (*n* = 14) were recruited and subjected to perioperatively continued sitagliptin intake (*n* = 7). For characterization of the immune profile, blood was drawn the first morning after surgery (i.e., 18 h postoperatively, Fig. 1A). Patients had a mean age of 67.1 ± 6.2 years and underwent coronary artery bypass or valvular surgery. There were no pre- and intraoperative differences between the groups except for lower preoperative creatine kinase-MB values and longer aortic clamping time in sitagliptin-medicated patients (Table 1). We first confirmed preoperative intake of sitagliptin resulted in reduced levels of DPP4 activity (Suppl. Figure 1 A). Assessment of immune profiles revealed that preoperative DPP4 inhibition resulted in substantial postoperative changes of several immune cell populations (Fig. 1B-I, Suppl. Figure 2 A-H).

**Table 1:** Pre- and perioperative patient characteristics

	Control (*n* = 7)	Sitagliptin (*n* = 7)	*P*-value
Age (years)	67.0 ± 5.7	65.3 ± 6.3	0.602
Sex (male)	6	7	1.000
Weight (kg)	94.7 ± 12.9	91.1 ± 19.4	0.692
Body mass index (kg/m^2^)	30.7 ± 3.9	28.3 ± 4.7	0.309
Body surface area (m^2^)	2.1 ± 0.2	2.1 ± 0.3	1.000
**Type of surgery**			
Coronary Artery Bypass Graft	5	5	1.000
Valvular surgery	2	2	1.000
Duration of surgery (min)	216.7 ± 34.7	241.0 ± 63.6	0.393
CPB-time (min)	107.4 ± 16.1	146.3 ± 52.5	0.103
Aortic-Clamping time (min)	64.6 ± 16.3	97.7 ± 32.2	**0.038**
Packed red blood cells	0.6 ± 1.5	0.9 ± 1.2	0.379
**Co-morbidities**			
Congestive heart failure	1	2	0.515
Arterial hypertension	5	7	0.127
Coronary artery disease	7	5	0.127
Peripheral artery disease	1	0	0.368
Atrial fibrillation	3	2	0.577
Previous coronary intervention	4	1	0.094
Asthma	1	0	0.299
COPD	1	1	1.000
Chronic kidney disease	6	6	1.000
Type II diabetes mellitus	7	7	-
Neurologic disorders	1	4	0.094
Psychiatric disorders	1	0	0.299
**Medication**			
Aspirin	3	3	1.000
Clopidogrel	2	0	0.127
Coumarins/DOACs	3	2	0.577
ß-blocker	7	5	0.127
ACE inhibitors	3	3	1.000
Angiotensin II receptor blocker	1	3	0.237
Angiotensin receptor-neprilysin inhibitor	2	0	0.127
Diuretics	4	4	1.000
Statins	4	5	0.577
Metformin	7	4	0.559
**Baseline laboratory values**			
Leukocytes (Thd/µL)	8.7 ± 3.0	8.9 ± 3.4	1.000
Creatine kinase (U/L)	135.3 ± 80.0	73.1 ± 34.9	0.252
Creatine kinase-MB (U/L)	21.8 ± 7.1	15.2 ± 3.1	**0.041**
Aspartate transaminase (U/L)	30.9 ± 16.2	29.7 ± 10.4	0.878
Alanine transaminase (U/L)	31.3 ± 17.8	34.7 ± 27.1	0.949
Creatinine (mg/dL)	1.0 ± 0.2	1.8 ± 1.8	0.398
Estimated glomerular filtration rate (CKD-EPI)	73.0 ± 14.6	59.5 ± 30.9	0.522
**Glucose levels** (mg/dL)			
After induction of anesthesia	143.9 ± 12	181.6 ± 22.1	0.161
POD1 max. glucose	199.3 ± 12.6	219 ± 12.9	0.291
POD1 min glucose	108.6 ± 10.3	122 ± 10.4	0.377

Significant *P*-values are indicated in bold

Within the CD4^+^ memory T-cell population, DPP4 inhibition was associated with mild to moderate changes of cluster abundance of over +/- 20% in 6 out of 15 clusters (cluster 2, 3, 9, 11, 14, 15) (Fig. 1C). Further, DPP4 inhibition favored a shift from differentiated CD4^+^ memory T-cells towards naïve CD4^+^ T-cells. For example, the proportion of recent thymic emigrant (RTE)-cells increased under DPP4 inhibition, whereas CD4^+^ CD45RO^+^ memory T-cells declined (Fig. 1C, Suppl. Figure 2 A). Using manual gating, we found that DPP4 inhibition increased the proportion of CD4^+^ secondary effector T helper 1-cells (TH1-cells) (CCR4^−^CCR6^−^CD183^+^ CD4^+^ memory T-cells), while reducing T helper 17-cells (TH17-cells) (CCR4^+^CCR6^+^CD183^−^ CD4^+^ memory T-cells) after surgery (Fig. 1C). CD146 (MCAM) expression required for extravasation and migration of lymphocytes to inflamed tissues was reduced [27] (Fig. 1C). Within the CD8^+^ memory T-cell population, DPP4 inhibition induced changes of more than 20% in 4 out of 11 clusters (cluster 4, 6, 7, 10) and the log_2_ fold change was ≥1 in cluster 10. Cells in cluster 4 and 10 particularly expressed CD27, a key costimulatory receptor in generation of T cell memory [28] (Fig. 1D). Sitagliptin-medicated patients additionally exhibited reduced proportions of naïve B-cells postoperatively (Fig. 1E).

Considering monocyte adhesion- and migration markers, DPP4 inhibition-induced changes in cluster abundance of more than 20% in 9 out of 10 clusters (all clusters except for cluster 8); log_2_ fold changes ≥1 were observed in 2 out of 10 clusters (cluster 2, 3) (Fig. 1F). Further, analysis revealed that several clusters with strong vascular cell adhesion molecule-1 (VCAM)−1 expression declined, whereas clusters with low or missing VCAM-1-expression increased (Fig. 1F). We additionally found that T-cell immunoglobulin mucin-3 (TIM-3), an inhibitory cell surface molecule negatively controlling the expansion of diverse immune cell types [29], was upregulated on all, but primarily on classical monocytes (Suppl. Figure 2B). Within pro-inflammatory monocyte markers, DPP4 inhibition-induced changes over 20% in 9 out of 12 clusters (cluster 2, 3, 5, 6, 7, 9, 10, 11, 12). Log_2_ fold changes showed a prominent decline in cluster 5 and 12, which were characterized by low CD14- and high CD16-expression (Fig. 1G). Thus, it can be assumed that CD14^dim^ CD16^+^ non-classical monocytes, recognized for TNF-α- and reactive oxygen species-production [30], declined under sitagliptin-treatment. Using manual gating of diverse monocyte-panels, we found that CD40-expressing - one of the most important costimulatory molecules for combined monocyte-T-cell-activation [31] - monocyte subsets were reduced, especially intermediate (CD14^+^CD16^int^) monocytes (Fig. 1H). Intercellular adhesion molecule-1 (ICAM-1)- and platelet endothelial cell adhesion molecule-1 (PECAM-1)-expression (CD31) was reduced in all subsets, but primarily in intermediate and non-classical monocytes (Fig. 1I). The proportion of all three monocyte subsets expressing CXC3R1 dropped (Suppl. Figure 2 C); in non-classical monocytes the proportion of CXC3R1-cells also declined (Suppl. Figure 2D). There were higher levels of classical and intermediate monocytes expressing homing-chemokine-receptor CCR7 (Suppl. Figure 2E). Moreover, classical and intermediate monocytes expressing scavenger receptors CD163 and CD206, both representing markers of anti-inflammatory monocyte population [32], were increasingly detectable (Suppl. Figure 2E). In addition, all three monocyte subsets showed reduced programmed cell death protein 1 (PD-1) receptor expression, an inhibitory receptor promoting self-tolerance of the immune system (Suppl. Figure 2F). DPP4 inhibition led to a reduction in circulating CD141^+^ myeloid dendritic cells, representing a subset of myeloid DCs (mDCs, Suppl. Figure 2G). In both CD56^dim^ and CD56^bright^ natural killer (NK) cells, the expression of a major activation receptor for NK cells, natural killer group 2 member D (NKG2D), was reduced (Suppl. Figure 2H). At 18 h post- surgery, there were only few differences in humoral cytokines and chemokines between patient groups (Suppl. Figure 3). Taken together, these data strongly suggest that perioperative DPP4 inhibition curbs innate and adaptive cellular immune responses during surgery.

### DPP4 inhibition modulates systemic inflammatory responses in mice

**Fig. 2:**
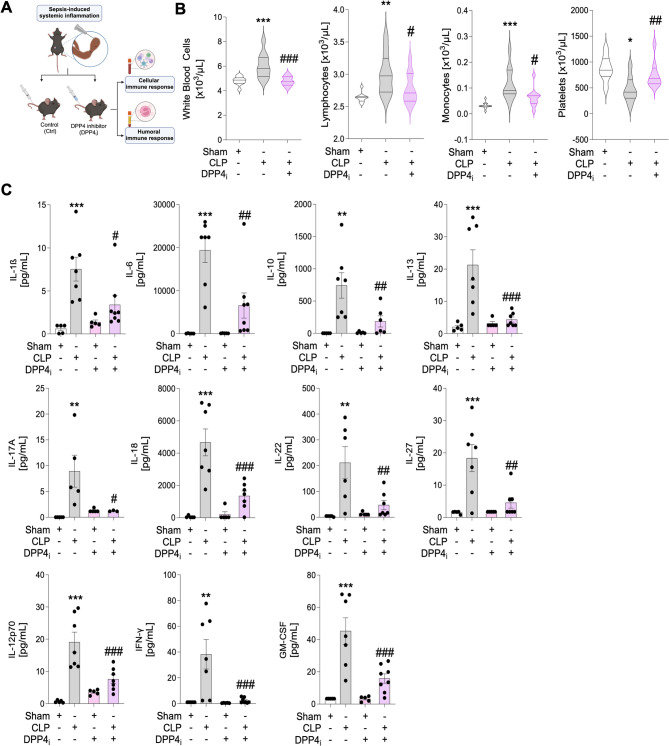
DPP4 inhibition modulates infection-associated inflammatory responses in mice. **A**: Schematic illustration of mice subjected to systemic inflammation by induction of a polymicrobial sepsis by cecal ligation and puncture (CLP). Created with BioRender.com. **B**: White blood cell, lymphocyte, monocyte and platelet count in murine blood 18 h after the induction of sepsis by CLP following the injection of the DPP4 inhibitor sitagliptin or vehicle serving as control intervention (*n* = 7–11 mice/group; mean ± SEM; one-way ANOVA/Bonferroni). **C**: Quantitative summary of significant changes in cytokine and chemokine levels 18 h after sepsis induction following administration of DPP4 inhibitor sitagliptin (*n* = 3–8 mice/group; mean ± SEM; one-way ANOVA/Bonferroni; Interleukin (IL), interferon (IFN), granulocyte-macrophage colony-stimulating factor (GM-CSF)). Asterisks indicate differences vs. sham- operated mice (*p < 0.05, **p < 0.01 and ***p < 0.001). Hashtags indicate statistically significant differences vs. septic mice (#*p* < 0.05, ##*p* < 0.01 and ###*p* < 0.001)

To further evaluate effects of DPP4 inhibition during systemic inflammation, we analyzed the impact of sitagliptin on mice subjected to severe systemic inflammation (i.e., sepsis due to polymicrobial peritonitis, Fig. 2A). Comparable to the changes to cellular immune responses in humans, DPP4 inhibition modulated immune cell populations in septic mice. DPP4 inhibition reduced white blood cell counts, especially of lymphocytes and monocytes and antagonized sepsis-induced thrombopenia (Fig. 2B). Of note, DPP4 inhibitor application reduced the concentrations of various pro-inflammatory mediators in murine blood 18 h after sepsis induction (Fig. 2C).

### DPP4 inhibition reduces vascular leakage in systemic inflammation

**Fig. 3:**
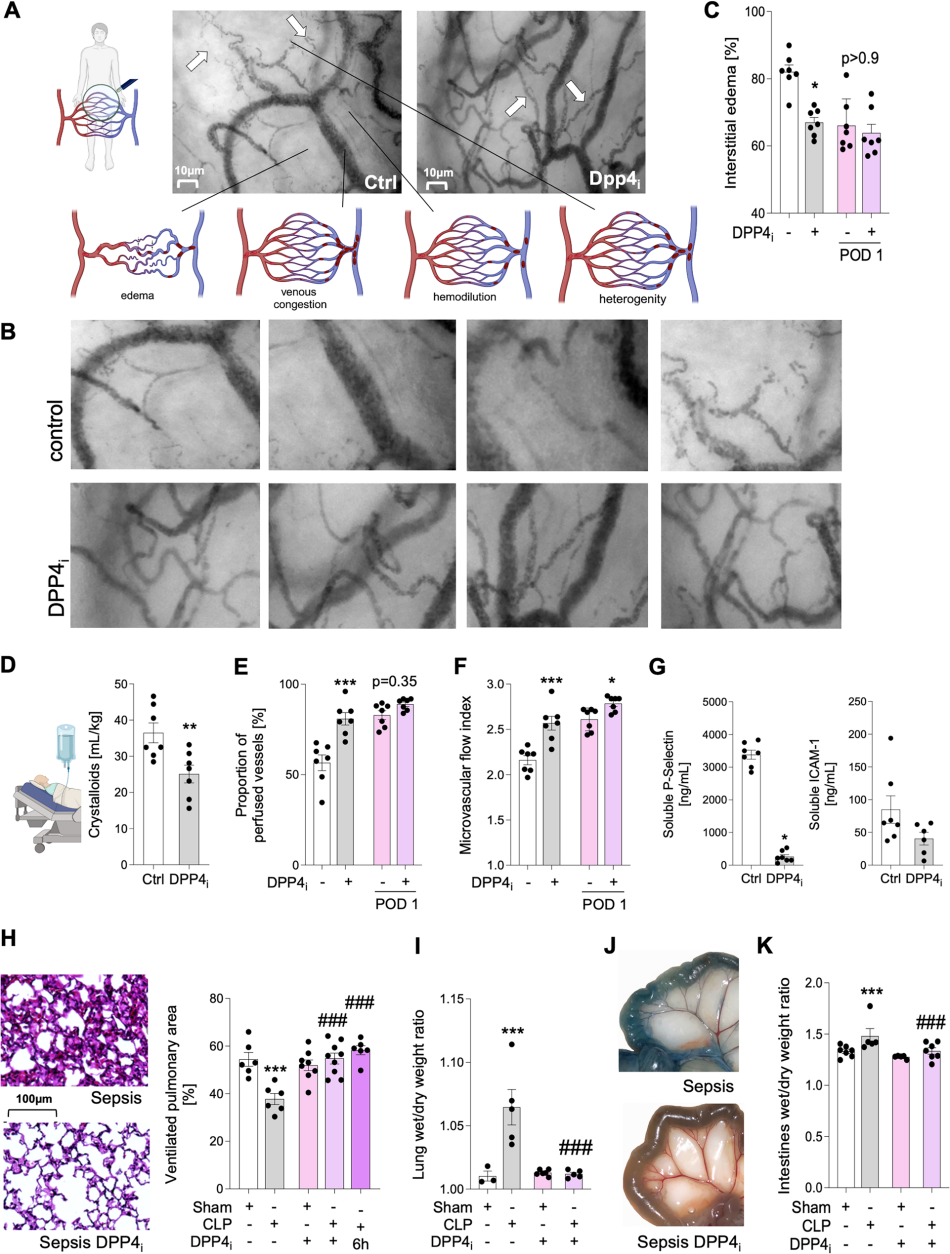
DPP4 inhibition reduces vascular leakage in systemic inflammation. **A**,** B**: Functional characteristics of the sublingual microcirculation assessed in patients with preoperative DPP4 inhibitor intake or without serving as controls. Arrows mark vessels with compromised or intact microcirculation. BioRender.com. **C**: Comparison of extent of edema analyzed by fluid area measurements between the groups immediately and on the first postoperative day (POD 1) (*n* = 7 patients/group; mean ± SEM; one-way ANOVA/Bonferroni). **D**: Total postoperative crystalloid volume requirements based on passive-leg-raise test (*n* = 7 patients/group; mean ± SEM; unpaired t-test). **E**: Comparison of the proportion of perfused vessels (PPV) between the groups immediately and on POD1 (*n* = 6–7 patients/group; mean ± SEM; unpaired t-test). **F**: Comparison of the microvascular flow index (MFI) immediately and on POD1 (*n* = 6–7 patients/group; mean ± SEM; unpaired t-test). **G**: Differences in blood concentrations of p-selectin and ICAM-1 18 h postoperatively (*n* = 7 patients/group; mean ± SEM; un- paired t-test). **H**: Pulmonary edema visualized by hematoxylin and eosin-stained micrographs of murine lungs 18 h after sepsis induction and injection of DPP4 inhibitor or vehicle. Quantitative summary of ventilated pulmonary area 18 h after sepsis induction following administration of DPP4 inhibitor sitagliptin directly and with 6 h time delay (*n* = 6–9 mice/group; mean ± SEM; one-way ANOVA/Bonferroni). **I**: Lung tissue edema of septic mice treated with DPP4 inhibitor or respective control, indicated as tissue wet/dry ratio 18 h after sepsis induction (*n* = 3–5 mice/group; mean ± SEM; one-way ANO-VA/Bonferroni). **J**: Representative photographs showing Evans blue content of murine intestines presenting albumin that extravasates during tissue edema formation. **K**: Tissue edema of murine intestines after sepsis induction and DPP4i, measured as tissue wet/dry ratio 18 h after sepsis induction (*n* = 5–7 mice/group; mean ± SEM; one-way ANOVA/Bonferroni). Scale bars represent 10 μm. Asterisks indicate differences vs. patient control group/sham-operated mice (*p < 0.05, **p < 0.01, ***p < 0.001 or p-value = as indicated). Hashtags indicate statistically significant differences vs. septic mice (###*p* < 0.001)

In patients, the systemic inflammatory response induced by surgical trauma is associated with dysfunction of the micro- and microvasculature [33]. To reveal whether altered perioperative inflammatory responses associated with DPP4 inhibitor intake translate into changes of vascular integrity, we assessed functional characteristics of the sublingual microcirculation, both immediately after surgery and on the first postoperative day (POD1) on the ICU. Directly after surgery, patients exhibited edema formation, signs of venous congestion, hemodilution and heterogeneity in capillary perfusion (Fig. 3A). In contrast, patients, who had received DPP4 inhibitors preoperatively, showed reduced areas of interstitial edema in the sublingual tissue (Fig. 3B-C), suggesting a more intact vascular barrier. This assumption was further supported by reduced postoperative intravenous fluid demands in DPP4 inhibitor-treated patients (Fig. 3D). Reduced interstitial edema in DPP4 inhibitor-treated patients further correlated with a higher PPV of blood vessels 6 h after surgery and 18 h later (Fig. 3E), as well as a higher MFI (Fig. 3F). In addition to functional microvascular parameters, DPP4 inhibition was associated with reduced soluble markers of endothelial cell damage, such as p-selectin and ICAM-1 postoperatively (Fig. 3G). We further dissected the effect of DPP4 inhibition on vascular barrier integrity in mice using a model of polymicrobial sepsis inducing severe systemic inflammation. 18 h after sepsis induction, mice exhibited fulminant pulmonary edema resulting in significant reduction in ventilated pulmonary area that is crucial for efficient gas exchange (Fig. 3H, I). Conversely, DPP4 inhibition by both sitagliptin and the selective DPP4 inhibitor K579, another DPP4 inhibitor approved for laboratory research, resulted in increase in ventilated pulmonary area (Fig. 3H; Suppl. Figure 4) and reduced fluid accumulation in the pulmonary interstitium (Fig. 3I). Importantly, DPP4 inhibition also applied 6 h after sepsis induction prevented from sepsis-induced pulmonary edema (Fig. 3H). In addition to murine lungs, vascular barrier protection upon DPP4 inhibition was also evident in other organs, such as the intestines, where we observed less visible tissue extravasation of Evans blue-bound albumin qualitatively (Fig. 3J) corresponding to differences in wet/dry ratio of intestines in septic vs. DPP4i-treated septic mice (Fig. 3K). These in vivo data suggest DPP4 inhibition is a potent means to ameliorate vascular barrier dysfunction during inflammation.

### DPP4 inhibition reduces vasoplegia and protects organ integrity

**Fig. 4:**
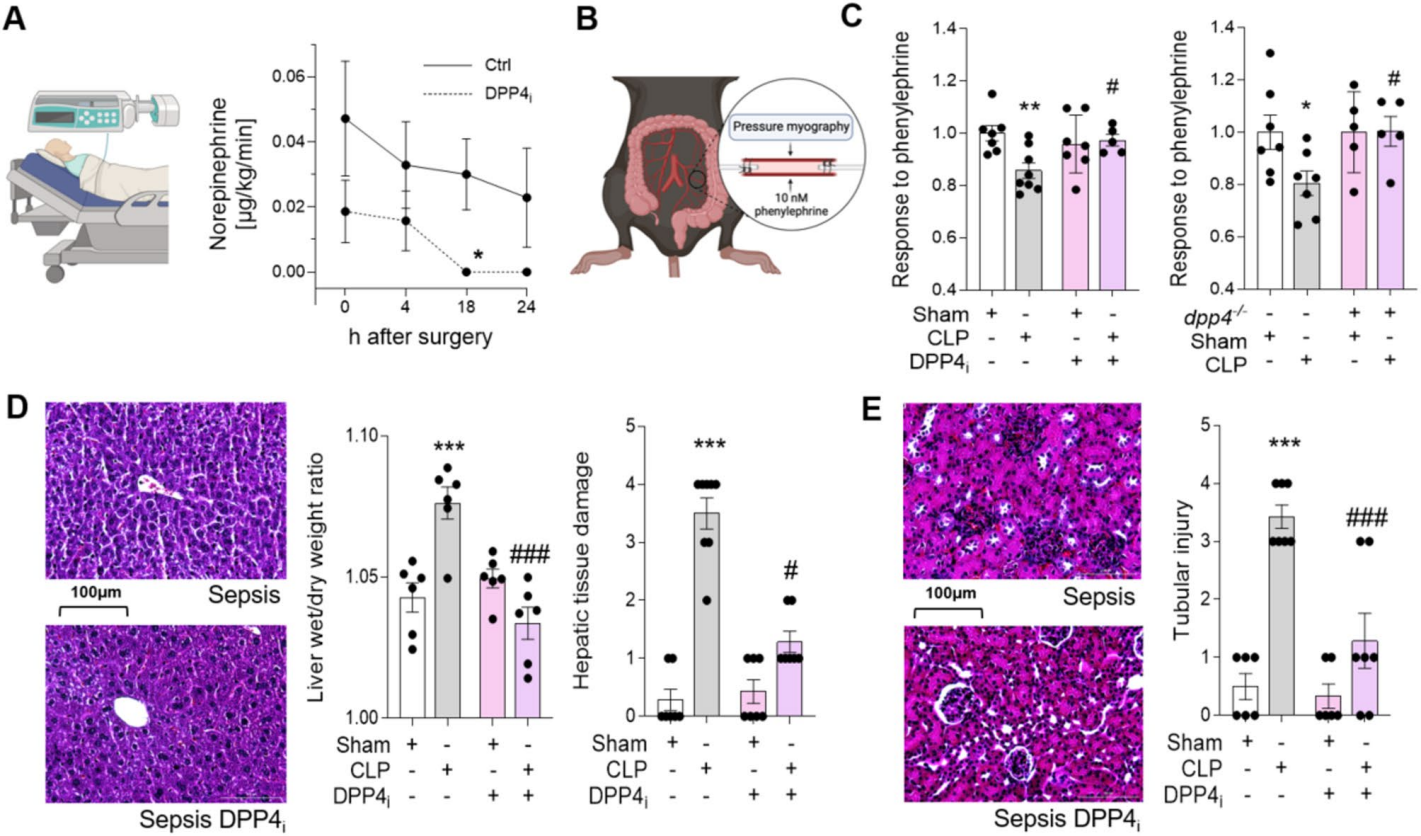
DPP4 inhibition reduces vasoplegia and protects organ integrity. **A**: Time course curve of norepinephrine requirements [µg/kg/min] within the first 24 h after surgery (*n* = 7 patients/group; mean ± SEM; Mann-Whitney-test). Graphic was created with BioRender.com. **B**,** C**: Response of murine third-order mesenteric resistance arteries to 10nM phenylephrine hydrochloride 18 h after sepsis induction in wild type mice subjected to DPP4 inhibition or control treatment (left panel) and in dpp4-/- mice (*n* = 5 mice/group; mean ± SEM; one-way ANOVA/Bonferroni). Graphic was created with BioRender.com. **D**: Representative pictures showing hepatic tissue edema and damage 18 h after CLP/sham surgery following treatment with DPP4 inhibitor or control. Liver tissue edema of septic mice treated with DPP4 inhibitor or respective control indicated as tissue wet/dry ratio (*n* = 6 mice/group; mean ± SEM; one-way ANOVA/Bonferroni). Quantitative summary of hepatic tissue damage (*n* = 3–8 mice/group; mean ± SEM; one-way ANOVA/Bonferroni). **E**: Representative pictures and quantitative summary of tubular injury 18 h after CLP/sham surgery following injection of DPP4 inhibitor sitagliptin (*n* = 3–5 mice/group; mean ± SEM; one-way ANOVA/Bonferroni). Scale bars of histological micrographs represent 100 μm. Asterisks indicate differences vs. patient control group/sham-operated mice (*p < 0.05, **p < 0.01 and ***p < 0.001). Hashtags indicate differences vs. septic mice (#*p* < 0.05 and ###*p* < 0.001)

In addition to capillary leakage, patients with a systemic inflammatory response often present vasoplegia requiring elevated doses of vasopressors for adequate maintenance of mean arterial blood pressure. We thus assessed parameters of vasoplegia in control- vs. DPP4 inhibitor-medicated patients. While control-treated patients mostly required postoperative vasopressor administration, none of the patients with DPP4 inhibitors required a postoperative vasopressor after 18 and 24 h post-surgery (Fig. 4A; Table 2). We further assessed the response to a vasopressive agent in septic mice by analyzing the response of murine mesenteric resistance arteries to phenylephrine ex vivo (Fig. 4B). Here, sepsis induced a prominent loss in vasopressor response (Fig. 4C). In contrast, application of DPP4 inhibition resulted in an augmented response to phenylephrine (Fig. 4C). In these experiments, mice deficient of DPP4 were used as additional controls showing comparable preservation of responses to vasopressors as DPP4 inhibitor-medicated wildtype mice (Fig. 4C). To assess whether vascular protection by DPP4 inhibition translates into preserved organ function during sepsis, we analyzed integrity of murine liver and kidney. In line with observations from murine lungs, septic mice exhibited interstitial fluid accumulation and damaged liver tissue, while these effects were diminished by DPP4 inhibition (Fig. 4D). Further, in murine kidneys, DPP4 inhibition ameliorated acute tubular injury (Fig. 4E). These findings suggest DPP4 inhibition reduces vasoplegia and is a potent measure for organ protection during systemic inflammatory response.

**Table 2:** Postoperative clinical data

	Control (*n* = 7)	Sitagliptin (*n* = 7)	*P*-value
At ICU admission			
Leukocytes (Thd/µL)	11.19 ± 1.4	10.07 ± 1.54	0.565
Creatine kinase (U/L)	310.86 ± 46.09	423.71 ± 66.64	0.25
Creatine kinase-MB (U/L)	42.43 ± 7.01	56.57 ± 9.9	0.142
CK-MB/CK (%)	13.43 ± 0.37	13.43 ± 0.81	0.599
Aspartate transaminase (U/L)	48.29 ± 6.26	64.71 ± 8.13	0.11
Alanine transaminase (U/L)	25.71 ± 5.14	35.14 ± 10.71	0.522
Creatinine (mg/dL)	0.9 ± 0.1	1.56 ± 0.58	0.199
Horovitz index (paO_2_/F_i_O_2_)	298.14 ± 44.69	272.43 ± 39.18	0.749
Cardiac output (L/min)	5.18 ± 0.48	4.56 ± 0.54	0.347
Lactate (mmol/L)	1.23 ± 0.26	1.26 ± 0.17	0.654
Norepinephrine (µg/kg/min)	0.47 ± 0.1	0.19 ± 0.01	0.152
**First postoperative day (POD1**,** 18 h after surgery)**			
Leukocytes (Thd/µL)	12.32 ± 0.93	11.16 ± 0.85	0.482
Creatine kinase (U/L)	446.14 ± 81.75	472.86 ± 72.97	0.848
Creatine kinase-MB (U/L)	34.71 ± 6.82	34 ± 6.14	0.949
CK-MB/CK (%)	8.14 ± 1.06	7.57 ± 1.21	0.561
Aspartate transaminase (U/L)	56 ± 8.64	69 ± 8.71	0.338
Alanine transaminase (U/L)	28.86 ± 6.24	36 ± 8.49	0.522
Creatinine (mg/dL)	0.87 ± 0.08	1.71 ± 0.64	0.070
Horovitz index (paO2/FiO2)	261.86 ± 40.72	288.86 ± 31.64	0.443
Cardiac output (L/min)	5.43 ± 0.34	5.16 ± 0.4	0.715
Lactate (mmol/L)	1.41 ± 0.5	1.06 ± 0.16	0.886
Norepinephrine (µg/kg/min)	0.03 ± 0.01	0 ± 0	**0.009**

Significant *P*-values are indicated in bold

### DPP4 inhibition modulates endothelial cell genomic profiles during sepsis

**Fig. 5:**
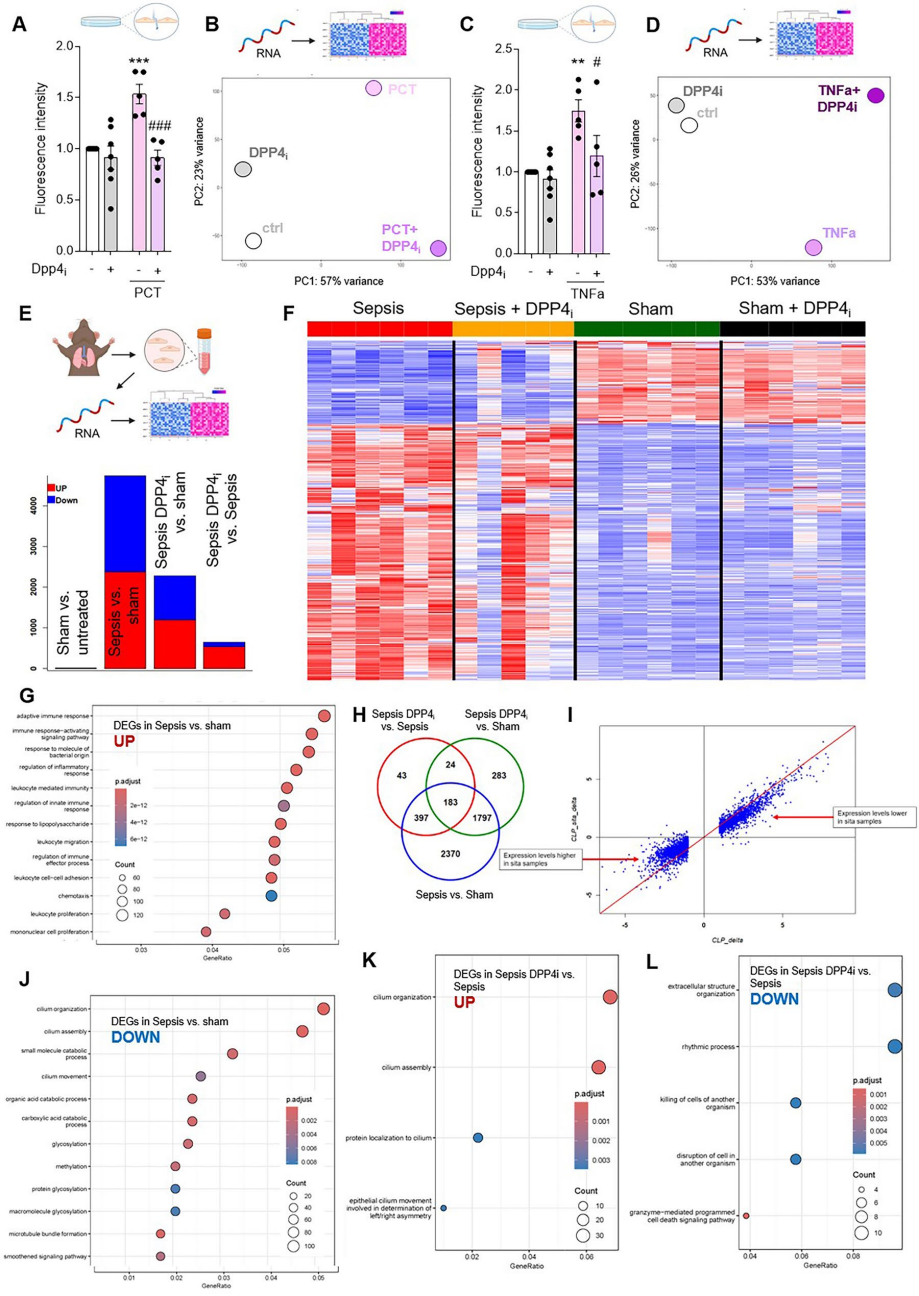
DPP4 inhibition modulates endothelial cell genomic profiles during sepsis. **A-D**: Effects of DPP4 inhibition on human pulmonary microvascular endothelial cell monolayer permeability upon exposure to DPP4 substrate procalcitonin (PCT) or TNF-alpha assessed in a two-chamber assay by fluorescence intensity of 70 kDa fluorescein isothiocyanate-labeled dextran macromolecules in lower chambers. Values are calculated relative (timesfold) to the mean of the control value defined as 1.0 (*n* = 5–9 independent experiments/group, one-way ANOVA/Bonferroni). Asterisks indicate differences vs. control (***p < 0.001). Hashtags indicate differences vs. respective PCT treatment (###*p* < 0.001). Principal component analysis (PCA) of RNA sequencing of endothelial cells exposed to procalcitonin with or without sitagliptin as DPP4 inhibitor. **E**: Endothelial cells were isolated from murine lungs (*n* = 5–6 mice/group) 18 h after cecal ligation and puncture (CLP) or sham surgery following treatment with DPP4 inhibitor or control. Endothelial RNA was subjected to bulk RNA sequencing. Summary of the number of differentially expressed genes (DEGs) in murine endothelial cells. **F**: Heatmap of expression levels of DEGs from contrasts. Values were scaled by row. red: up-regulated DEGs, blue: down-regulated DEGs. Each column in the heatmap represents one mouse. **G**,** H**: Top up- and downregulated genes related to gene ontology in septic vs. non-septic mice. **I**,** J**: VENN diagram deciphering the number of DEGs from the same contrasts and scatterplot showing sitagliptin results in less regulation of genes induced by sepsis. **K**,** L**: Up- and downregulated genes in septic mice subjected to treatment with sitagliptin in contrast to septic mice. Graphics were created with BioRender.com

To mechanistically verify DPP4-dependency of pro-inflammatory signaling in endothelial cells, we analyzed the endothelial cell permeability-inducing effects of DPP4 substrate PCT and TNF-alpha in the presence or absence of DPP4 inhibition. PCT, as well as TNF-α were validated to induce leakage of an endothelial monolayer in vitro. This effect was almost abolished when endothelial DPP4 was inhibited prior to treatment (Fig. 5A, C). Correspondingly, RNA-sequencing of endothelial cells verified shift in genomic expression profiles induced by procalcitonin that was modulated by DPP4 inhibition (Fig. 5B, D). We next aimed to verify the changes that translate from immunomodulation by DPP4 inhibition to the vasculature at the transcriptomic level in vivo. Since the lung is one of the most susceptible organs to systemic inflammation and we had verified functional changes to the pulmonary vascular barrier (Fig. 3H, I), we subjected pulmonary endothelial cells from septic mice to RNA sequencing 18 h after sepsis induction (Fig. 5E). Sepsis per se induced changes to more than 4,000 differentially expressed genes (DEGs) in the murine endothelium (Fig. 5E) with half of them upregulated upon DPP4 inhibition, while half of them were downregulated. Analysis of endothelial transcriptome profiles in mice revealed a shift of towards lower expression levels by DPP4 inhibition during sepsis compared to untreated septic mice (Fig. 5F). Sepsis revealed a strong upregulation of genes involved in the orchestration of inflammatory cells and downregulation of genes involved in vascular barrier maintenance and integrity such as cilia [34, 35] and their organization and downregulation of genes involved in cellular metabolism (Fig. 5G, H). Of note, application of DPP4 inhibitors during sepsis primarily reduced the number of genes regulated, rather than inducing other sets of genes (Fig. 5I, J). Application of the DPP4 inhibitor sitagliptin specifically restored the sepsis-induced downregulation of cilium organization and cell structure and downregulated pathways involved in endothelial responses to inflammation (Fig. 5K, L).

## Discussion

DPP4 modifies N-termini that regulate receptor-preferences and activity of a vast majority of humoral substrates involved in systemic inflammation. Major surgery is one of the most potent inducers of a sterile inflammatory response associated with postoperative capillary leakage and vasoplegia. We here show that the preoperative intake of a DPP4 inhibitor results in changes to the phenotype of innate and adaptive immune cell populations in patients 24 h after cardiac surgery, indicating a summative effect of DPP4 on its substrates involved in the characteristics of inflammation. For example, DPP4 inhibition resulted in reduced activation of monocytes, dendritic and natural killer cells. T-cell traits were shifted towards younger and naïve subsets in addition to lower numbers of memory T-cells associated with rapid pro-inflammatory responses, extravasation and tissue migration properties. In mice, DPP4 inhibition partially reverted sepsis-induced leukocytosis, thrombopenia and cytokine concentrations. In both humans and mice, these changes to the characteristics of the systemic inflammatory response were associated with augmented vascular integrity and function we believe is a suitable readout for the overall inflammatory response. Most strikingly, we found that sepsis-induced gene activation in the endothelium was massively reduced. In mice, these findings also correlated with measures of reduced inflammation-induced organ damage.

The results reported here suggest that the summative effect of DPP4 inhibition on surgery- and sepsis-induced systemic inflammation is mitigation of its severity. DPP4 has many substrates with long established, prominent roles in systemic inflammation, such as IL-6, IL-1, IL-8, IL-10, interferon (IFN)-γ, TNF-α, granulocyte-macrophage colony-stimulating factor (GM-CSF), monocyte chemoattractant protein-1(MCP-1), macrophage inflammatory protein-1 (MIP-1) α, interferon gamma-induced protein-10 (IP-10) [36]. However, we did not observe any differences in total cytokine levels between the sitagliptin-treated and control group in the human study. However, only assessment of the N-terminal structure rather than their mere concentration of the DPP4 substrates involved can proof the direct mechanism of action of DPP4 inhibition [37]. Based on the functionality of DPP4 and the fact that only little is known about the differential activity on the respective cytokine receptors with regards to the presence or absence of the N-terminal dipeptide, we assume that we here observe the result of a modulation of a multitude of single pathways involved in the composition of the inflammatory response. Future studies will need to shed light on the functional relevance of DPP4-mediated N-terminal truncation on cytokine activity. However, some of these putative pathways can be a priori identified as likely contributors. For example, DPP4 inhibitors are clinically licensed for lowering glucose levels by prolonging the half-life and thus the action of the glucagone-like peptide 1 (GLP-1) on the GLP-1 receptor. Reduction of GLP-1 receptor agonist activity has been shown to exert multiple anti-inflammatory effects in conditions like diabetes, neurodegenerative diseases, as Parkinson’s disease, and inflammatory bowel diseases [38]. Although the effects of GLP-1 receptor antagonization has been investigated only scarcely in the setting of acute systemic inflammation, it is likely that the prolonging of GLP-1 half-life by DPP4 inhibitor application contributes to the beneficial effects of DPP4 inhibition observed here. DPP4 also cleaves the high mobility group box 1 (HMGB1) protein, a strong mediator of trauma-induced innate immune responses [2]. DPP4 inhibitors have been shown to exert beneficial effects by specifically modulating HMGB1 activity [39], thus reducing pro-inflammatory HMGB1 signaling via toll-like receptors (TLRs) [40] that in turn crucially mediates organ injury during trauma [41]. In addition, modulation of mediators directly acting on the endothelium may additionally contribute to the effects of DPP4 inhibitors. In this regard, we have recently shown that PCT induces vascular leakage in a DPP4-dependent manner [14] and here now provide evidence for differential effects on gene expression in human endothelial cells of procalcitonin in the presence vs. absence of DPP4 inhibitors. As DPP4 is ubiquitously expressed on endothelial cells and thus present not only in every in vivo, but also in vitro system, it may be assumed that the effect of DPP4 mediated-cleavages of substrates acting on the endothelium is underestimated when dissecting the effects of humoral mediators on endothelial function and integrity.

Acute inflammatory syndromes as observed in sepsis are associated with exceptionally high and – over the past decades – almost unchanged mortality due to organ dysfunction and failure [42]. Acute inflammation is accompanied by strong phenotypes of vascular dysfunction such as vasoplegia and capillary leakage syndrome that both become clinically evident quickly during the course of disease and are clearly associated with adverse outcome [1]. Preservation of vascular integrity is thus considered a frontside approach to reduce inflammation-induced organ damage. However, anti-inflammatory or immune-modulating strategies targeting single pathways, such as TNF-α, TLRs or interleukins, similarly as immuno-suppressive therapies have so far all failed to translate into improvement of clinical outcomes [43]. Thus, a broad, unspecific and multifactorial approach that modulates immune responses costum-tailored to the individual responses in an individual patient may display a novel, promising approach to curb dysregulated immune responses that translates into augmented vascular function, organ integrity and finally into improved outcomes. We here show that DPP4 inhibitors during acute systemic inflammation in humans and mice are clearly associated with augmented and preserved vascular function. Specifically, we observed robust effects on vasoplegia and capillary leakage in cardiac surgery patients that further translate into different entities of microcirculatory dysfunction previously identified as essential to inflammation-induced vascular pathology [44]. Not only cardiac surgery but almost all kinds of major surgery associated with extensive tissue trauma are accompanied by a systemic inflammatory response. Surgery is also associated with an unacceptably high rate of post-operative organ dysfunction, such as delirium or long-term cognitive deficits, pulmonary and renal dysfunction and myocardial injury [45]. These complications accumulate to numbers of perioperative mortality (i.e., within 30 days of surgery) that make up the third leading cause of death world-wide [46] and calls for novel strategies to ameliorate surgery-induced organ dysfunction [47]. Causally, the immune response to surgical trauma and stress is clearly identified as a major culprit in mediating perioperative damage to tissues, most likely via affecting vascular integrity.

In this study, we show that DPP4 inhibitors that potentially modulate a multitude of humoral mediators involved in orchestrating inflammatory responses, potently curb dysregulated immune responses to surgical trauma in patients and septic mice and preserve vascular integrity. A central limitation to this study is the fact that we only investigated patients with known type II diabetes mellitus as it is known that diabetes itself is a chronic low-grade inflammatory disease and diabetic patients may display altered immune responses to the surgical trauma. In addition, the number of studied patients is small, which leads to insufficient statistical power. Here, larger clinical trials will need to dissect the consequence of DPP4 inhibition on organ function after cardiac surgery. A further limitation of the findings reported here is the limited translational implication due to the use of two distinct “models”, i.e., human sterile, surgical inflammation and sepsis in mice. However, we aimed to validate the beneficial effects in a preclinical model of severe systemic inflammation associated with an overt phenotype of capillary leakage and organ damage. In turn, beneficial effects of DPP4i are also likely in septic patients.

## Conclusions

Our data suggest that inhibition of DPP4 during systemic inflammation may display a novel, safe, potent and thus key approach to contain perioperative immune responses and those related to systemic infection that translate in to organ protection and improved outcomes. In future studies, DPP4 inhibition may thus merit further clinical evaluation in settings of acute systemic inflammation.

## Supplementary Information

Supplementary Material 1: Figure 1. A: Postoperative DPP4 enzyme activity 6h after cardiac surgery measured in human serum in the DPP4i-group compared to the control-group (n=7 patients/group; mean ± SEM; unpaired t- test). Asterisk indicates difference vs. control group (*p<0.05). Figure 2. **A**: Percentage of recent thymic emigrant-cells (RTE-cells) among total lymphocytes. B: T-distributed stochastic neighbor embedding (t-SNE) map and mean fluorescence intensity of T-cell immunoglobulin mucin-3 (TIM-3) positive monocytes. C: Mean fluorescence intensity (MFI) of C-X3-C Motif Chemokine Receptor 1 (CX3CR1) positive monocytes. D: t-SNE map and mean fluorescence intensity of CX3CR1 positive non-classical monocytes. **E**: Mean fluorescence intensity of C-C chemokine receptor type 7 (CCR7), CD163 and CD206 positive monocytes. F: t-SNE map and mean fluorescence intensity of Programmed cell death protein 1 (PD-1) positive monocytes. G: Myeloid dendritic cells (mDCs) among total PBMCs.H: Quantity of natural killer group 2 member D+ (NKG2D+) NK cells. All figures: Diabetic patients vs. diabetic patients with DPP4 inhibitor treatment 18h after surgery; n=7 patients/group; mean ± SEM; unpaired t- test. Asterisks indicate differences vs. control (*p<0.05), ns=not significant vs. control, or p- value=as indicated vs. control group. Figure 3. A: Quantitative summary of changes in cytokine and chemokine levels in diabetic control vs. diabetic patients with DPP4 inhibitor treatment 18h after cardiac surgery (Granulocyte-macrophage colony-stimulating factor (GM-CSF), interferon (IFN), interleukin (IL), interferon gamma-induced protein-10 (IP-10), monocyte chemoattractant protein-1 (MCP-1), Macrophage inflammatory protein-1 (MIP-1), tumor necrosis factor (TNF), interferon (IFN)). All figures: n=7 patients/group; mean ± SEM; unpaired t- test. Asterisk indicates difference vs. control group (*p<0.05) or p-value=as indicated vs. control group. Figure 4. A: Pulmonary edema visualized by hematoxylin and eosin-stained micrographs of murine lungs 18h after sepsis induction and injection of vehicle or DPP4 inhibitor K579. B: Quantitative summary of ventilated pulmonary area 18h after sepsis induction following administration of the DPP4 inhibitor sitagliptin after cecal ligation and puncture (CLP)/sham surgery (n=6-9 mice/group; mean ± SEM; one-way ANOVA/Bonferroni). Asterisks indicate difference vs. sham-operated mice (**p<0.01). Hashtags indicate difference vs. septic mice (###p<0.001).

## Data Availability

No datasets were generated or analysed during the current study.

## Abbreviations

Bh-SNE: Barnes-Hut stochastic neighbor embedding
Ca: Calcium
CaCl_2_: Calcium chloride
CCR7: C-C chemokine receptor type 7
CX3CR1: C-X3-C Motif Chemokine Receptor 1
CD: Cluster of differentiation
CK: Creatine kinase
CLP: Cecal ligation and puncture
COPD: Chronic obstructive pulmonary disease
CPB: Cardiopulmonary bypass
Ctrl: Control
DEGs: Differentially expressed genes
DNAM-1: DNAX Accessory Molecule-1
DOACs: Direct oral anticoagulants
DPP4: Dipeptidyl peptidase-4
EGTA: Ethylene glycol-bis(β-aminoethyl ether)-N,N,N′,N′-tetraacetic acid
ELISA: Enzyme-linked immunosorbent assay
EM: Effector memory
FACS: Fluorescence-activated cell sorting
FCS: Fetal calf serum
FITC: Fluorescein isothiocyanate
GIP: Gastric inhibitory polypeptide
GLP-1: Glucagon like peptide-1
GM-CSF: Granulocyte-macrophage colony-stimulating factor
HEPES: 4-(2-hydroxyethyl)-1-piperazineethanesulfonic acid
HMGB1: High mobility group box 1
HUVECs: Human umbilical vein endothelial cells
HPMECs: Human pulmonary microvascular endothelial cells
ICAM-1: Intercellular adhesion molecule-1
IFN: Interferon
IL: Interleukin
IP-10: Interferon gamma-induced protein-10
KCl: Potassium chloride
MCP-1: Monocyte chemoattractant protein-1
MFI: Microvascular flow index
MgCl_2_: Magnesium chloride
MIP-1: Macrophage inflammatory protein-1
mDCs: Myeloid dendritic cells
Na: Sodium
NaCl: Sodium chloride
NK: Natural killer
NKG2D: Natural killer group 2 member D
PBMC: Peripheral Blood Mononuclear Cell
PBMCs: Peripheral Blood Mononuclear Cells
PCA: Principal component analysis
PCT: Procalcitonin
PD-1: Programmed cell death protein-1
PECAM-1: Platelet endothelial cell adhesion molecule-1
PMA: Polymethacrylate
POD: Postoperative day
PPV: Proportion of perfused vessels
RPMI: Roswell Park Memorial Institute
RTE: Recent thymic emigrant
SEM: Standard error of mean
TH1-cells: T helper 1-cells
TH17-cells: T helper 17-cells
TIM-3: T-cell immunoglobulin mucin-3
TLRs: Toll-like receptors
TNF: Tumor necrosis factor
t-SNE: T-distributed stochastic neighbor embedding
VCAM-1: Vascular cell adhesion molecule-1
