# Supplementary figures and images for: DPP4 inhibition curbs systemic inflammation
Source: Crit Care. 2025 Aug 15;29:359. doi: 10.1186/s13054-025-05599-x (PMC12357438; doi:10.1186/s13054-025-05599-x)

Suppl. Fig. 1

A

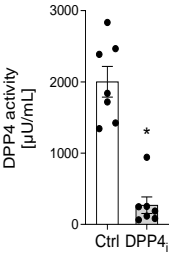

Suppl. Fig. 2

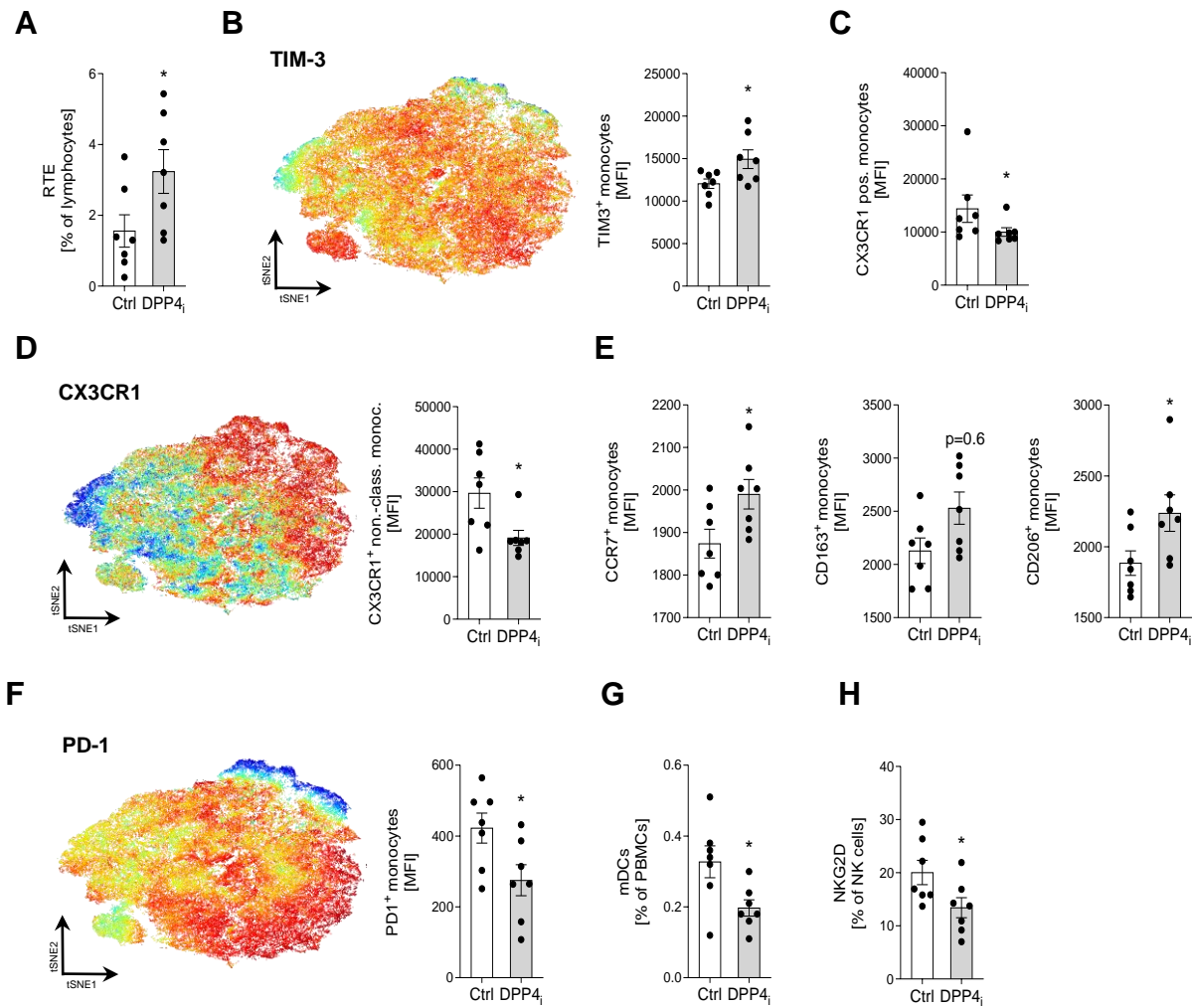

Suppl. Fig. 3  
A

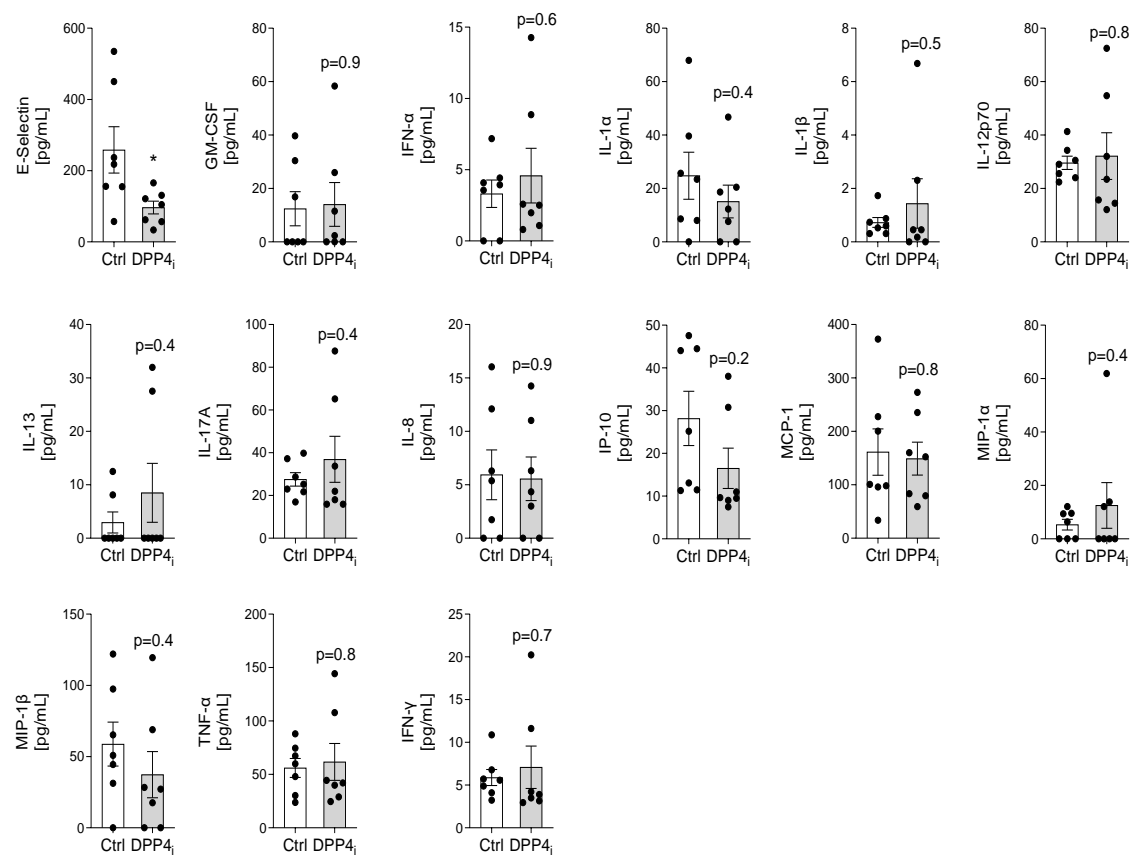

Suppl. Fig. 4

A

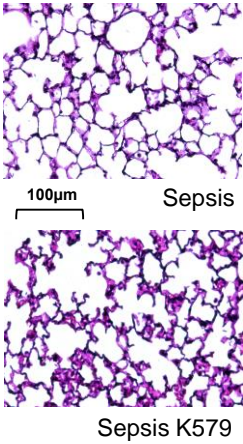

B

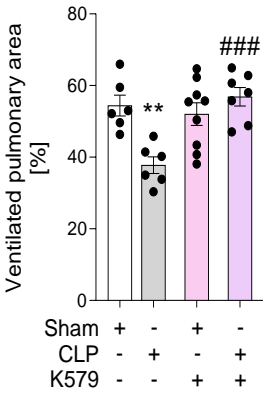

Supplement: Supplementary file 1 — Supplementary Material 1: Figure 1. A: Postoperative DPP4 enzyme activity 6h after cardiac surgery measured in human serum in the DPP4i-group compared to the control-group (n=7 patients/group; mean ± SEM; unpaired t- test). Asterisk indicates difference vs. control group (*p<0.05). Figure 2. A: Percentage of recent thymic emigrant-cells (RTE-cells) among total lymphocytes. B: T-distributed stochastic neighbor embedding (t-SNE) map and mean fluorescence intensity of T-cell immunoglobulin mucin-3 (TIM-3) positive monocytes. C: Mean fluorescence intensity (MFI) of C-X3-C Motif Chemokine Receptor 1 (CX3CR1) positive monocytes. D: t-SNE map and mean fluorescence intensity of CX3CR1 positive non-classical monocytes. E: Mean fluorescence intensity of C-C chemokine receptor type 7 (CCR7), CD163 and CD206 positive monocytes. F: t-SNE map and mean fluorescence intensity of Programmed cell death protein 1 (PD-1) positive monocytes. G: Myeloid dendritic cells (mDCs) among total PBMCs.H: Quantity of natural killer group 2 member D+ (NKG2D+) NK cells. All figures: Diabetic patients vs. diabetic patients with DPP4 inhibitor treatment 18h after surgery; n=7 patients/group; mean ± SEM; unpaired t- test. Asterisks indicate differences vs. control (*p<0.05), ns=not significant vs. control, or p- value=as indicated vs. control group. Figure 3. A: Quantitative summary of changes in cytokine and chemokine levels in diabetic control vs. diabetic patients with DPP4 inhibitor treatment 18h after cardiac surgery (Granulocyte-macrophage colony-stimulating factor (GM-CSF), interferon (IFN), interleukin (IL), interferon gamma-induced protein-10 (IP-10), monocyte chemoattractant protein-1 (MCP-1), Macrophage inflammatory protein-1 (MIP-1), tumor necrosis factor (TNF), interferon (IFN)). All figures: n=7 patients/group; mean ± SEM; unpaired t- test. Asterisk indicates difference vs. control group (*p<0.05) or p-value=as indicated vs. control group. Figure 4. A: Pulmonary edema visualized by hemat [file 13054_2025_5599_MOESM1_ESM.pdf]
